# Application of Genomic Selection at the Early Stage of Breeding Pipeline in Tropical Maize

**DOI:** 10.3389/fpls.2021.685488

**Published:** 2021-06-28

**Authors:** Yoseph Beyene, Manje Gowda, Paulino Pérez-Rodríguez, Michael Olsen, Kelly R. Robbins, Juan Burgueño, Boddupalli M. Prasanna, Jose Crossa

**Affiliations:** ^1^International Maize and Wheat Improvement Center (CIMMYT), Nairobi, Kenya; ^2^Colegio de Postgraduados, Montecillo, Mexico; ^3^School of Integrative Plant Science-Plant Breeding and Genetics Section, Cornell University, Ithaca, NY, United States; ^4^International Maize and Wheat Improvement Center (CIMMYT), Texcoco, Mexico

**Keywords:** early-stage testing, genomic selection, prediction accuracy, tropical maize, GBLUP

## Abstract

In maize, doubled haploid (DH) line production capacity of large-sized maize breeding programs often exceeds the capacity to phenotypically evaluate the complete set of testcross candidates in multi-location trials. The ability to partially select DH lines based on genotypic data while maintaining or improving genetic gains for key traits using phenotypic selection can result in significant resource savings. The present study aimed to evaluate genomic selection (GS) prediction scenarios for grain yield and agronomic traits of one of the tropical maize breeding pipelines of CIMMYT in eastern Africa, based on multi-year empirical data for designing a GS-based strategy at the early stages of the pipeline. We used field data from 3,068 tropical maize DH lines genotyped using rAmpSeq markers and evaluated as test crosses in well-watered (WW) and water-stress (WS) environments in Kenya from 2017 to 2019. Three prediction schemes were compared: (1) 1 year of performance data to predict a second year; (2) 2 years of pooled data to predict performance in the third year, and (3) using individual or pooled data plus converting a certain proportion of individuals from the testing set (TST) to the training set (TRN) to predict the next year's data. Employing five-fold cross-validation, the mean prediction accuracies for grain yield (GY) varied from 0.19 to 0.29 under WW and 0.22 to 0.31 under WS, when the 1-year datasets were used training set to predict a second year's data as a testing set. The mean prediction accuracies increased to 0.32 under WW and 0.31 under WS when the 2-year datasets were used as a training set to predict the third-year data set. In a forward prediction scenario, good predictive abilities (0.53 to 0.71) were found when the training set consisted of the previous year's breeding data and converting 30% of the next year's data from the testing set to the training set. The prediction accuracy for anthesis date and plant height across WW and WS environments obtained using 1-year data and integrating 10, 30, 50, 70, and 90% of the TST set to TRN set was much higher than those trained in individual years. We demonstrate that by increasing the TRN set to include genotypic and phenotypic data from the previous year and combining only 10–30% of the lines from the year of testing, the predicting accuracy can be increased, which in turn could be used to replace the first stage of field-based screening partially, thus saving significant costs associated with the testcross formation and multi-location testcross evaluation.

## Introduction

Breeding improved maize germplasm with tolerance to multiple abiotic and biotic stresses that occur in the tropics has been a major objective of the International Maize and Wheat Improvement Center (CIMMYT) since the 1960's (Vasal et al., 1999; Bänziger et al., 2006; Prasanna et al., 2021). CIMMYT's Eastern Africa maize breeding program has effectively released several high-yielding stress-tolerant hybrids and improved open-pollinated varieties suitable for target populations of environments in this region (Beyene et al., 2017b; Cairns and Prasanna, 2018; Worku et al., 2020). Among the various conventional breeding methods, pedigree breeding and the doubled haploids (DH) have been extensively used to develop fixed inbred lines with good *per se* performance and general combining abilities under stress and non-stress conditions. The recurrent selection method was widely used to increase the frequency of favorable alleles and maintain genetic variability under drought and optimum moisture conditions (Bolaños and Edmeades, 1996).

Currently, most of CIMMYT's maize breeding efforts are devoted to developing elite lines from biparental populations. Since the inception of the hybrid maize breeding program at CIMMYT in the mid-1980's, a total of 615 elite inbred lines have been released as CIMMYT Maize Lines (CMLs), which are international public goods (CIMMYT Global Maize Program, 2021). The CMLs can be accessed freely through the standard material transfer agreement and are used worldwide (Braun et al., 2010; Wu et al., 2016). These inbred lines have been used by maize breeders from CIMMYT, national agricultural research, and private seed companies to develop high-yielding and stress tolerance hybrids adapted to the different agro-ecologies of SSA (Bänziger et al., 2006; Beyene et al., 2017b; Makumbi et al., 2018; Prasanna et al., 2020; Worku et al., 2020).

The DH technology has been used in the CIMMYT maize breeding programs since 2012, gradually replacing conventional inbreeding to derive inbred lines (Prasanna et al., 2012, 2021). The establishment of maize DH facilities at CIMMYT in Mexico (2010–2011) and Kenya (2013) using tropicalized inducer lines (TAILs) has enabled large-scale development and utilization of DH lines in tropical maize breeding programs in both Latin America and Africa (Chaikam et al., 2019). Since 2011, CIMMYT has developed more than 400,000 DH lines from 1,280 diverse maize populations primarily targeted for mid-altitude/subtropics and lowland tropics of Africa and Latin America. Tropical DH lines with superior characteristics for *per se* performance (Worku et al., 2016; Beyene et al., 2017a), combining ability for stress tolerance (Beyene et al., 2013, 2017a; Ertiro et al., 2017; Sserumaga et al., 2018), and resistance to maize lethal necrosis (MLN) (Beyene et al., 2017b). Several DH-based elite maize hybrids from CIMMYT have also been released by partners in eastern and southern Africa (Beyene et al., 2017a).

Inbred line development starts with selecting parents possessing desirable traits, followed by the formation of segregating populations that allow for the selection of individuals possessing trait combinations relevant to target product profiles delineated for each breeding hub through recombination of novel alleles. Culling was also practiced opportunistically on disease resistance and lodging. A large number of selected candidates are tested on a single tester in relatively few environments during the initial testing phase, while a smaller number of selected candidates are tested on two or more testers in a larger number of environments in later testing phases. The experimental hybrids are evaluated under abiotic stresses and in the absence of acute stress to develop hybrids with stable performance across stressful and favorable growing conditions.

Currently, phenotyping accounts for the largest percentage of operational costs associated with breeding. Until recently, maize breeders at CIMMYT depended mostly on phenotypic selection to choose parents in the hybrid breeding program. The ability to select partially based on genotypic data while maintaining or improving genetic gains achieved using phenotypic selection alone can result in a significant cost saving.

Genomic selection (GS, Meuwissen et al., 2001) is being increasingly used in animal and plant breeding programs to predict the breeding value of untested genotypes using phenotypic and genotypic data from related genotypes. Beyene et al. (2015) reported that the average genetic gain per year in tropical maize grain yield using the GS approach was three times higher than that of conventional pedigree-based phenotypic selection (PS) in drought-stressed environments. A similar finding was reported by Vivek et al. (2017), in which the genetic gain in grain yield under drought using GS was up to 43% higher than the advanced version using conventional PS.

The effectiveness of GS is a product of the quality of the training population (TRN), with both genotypic and phenotypic data used to estimate the marker effects in the predicted population (TST). The strategy of test-half-and-predict-half based on marker data has been piloted in specific product profiles in eastern and southern Africa, as well as in Latin America, with highly encouraging results (Beyene et al., 2019; Santantonio et al., 2020; Wang et al., 2020; Atanda et al., 2021). The main objective of this study was to evaluate the potential of genomic prediction using breeding data from 1 year to predict the performance of phenotypically untested lines at an early testing stage to directly advance the best selection candidates to a second-year equivalent phenotypic trial, saving a year in the process of developing new elite hybrids and high potential breeding parents. We used data from a total of 3,068 lines genotyped using repetitive Amplification Sequencing (rAmpSeq) markers and phenotyped as test crosses over 3 years under well-watered (WW) and water-stressed (WS) conditions in SSA. The DH lines are from CIMMYT's Africa maize breeding program and are being used to develop hybrids adapted to rainfed, mid-altitude environments. The objectives were to (1) evaluate GS prediction abilities using historical breeding data to predict untested lines of the subsequent breeding year; and (2) compare with the predictive abilities when a selected portion of individuals from the testing set (or year) are converted into the training set.

## Materials and Methods

### Plant Material

We used testcross hybrids from the ongoing CIMMYT's eastern Africa maize breeding program. A total of 3,068 DH lines were used in this study, derived from 54 bi-parental populations, and the test crosses were evaluated in 2017, 2018, and 2019. The 54 bi-parental populations were obtained by crossing elite lines with drought tolerance and other farmer-preferred traits with satisfactory combining abilities. These populations represented the eastern Africa mid-altitude germplasm, and the developing hybrids were targeted for mid-altitude environments of eastern and southern Africa (Beyene et al., 2013).

### Field Experiments

Each year, each selected DH line was crossed with a single tester from the opposite heterotic group and phenotyped in WW and WS environments. The number of hybrids (trials) planted in 2017, 2018, and 2019 was 923 (14), 1,423 (34), and 722 (17), respectively. Four to six common checks connected the trials each year, including benchmark commercial hybrids and CIMMYT internal genetic gain checks. Each trial was planted in an alpha-lattice design with two replications per entry. The WW experiments were conducted during the rainy season (March to July), applying supplemental irrigation. The managed drought stress experiments were conducted during the dry (rain-free) season (June to October), and irrigation was suspended 2 weeks before flowering until harvest. Each entry was planted in two rows of 5 m long. The rows were spaced 0.75 m apart, and the space between hills was 0.25 m. Two seeds per hill were initially planted, and 3 weeks after emergence, thinned to one plant per hill to obtain a final plant population density of 53,333 plants/ha. Fertilizers were applied at 60 kg N and 60 kg P2O5/ha, as recommended for the area. Nitrogen was applied at planting and 6 weeks after emergence. Fields were kept free of weeds by hand weeding. Grain yield (GY, tons ha^−1^), anthesis date (AD, days), and plant height (PH, cm) traits were recorded. Plots were manually harvested, and GY was corrected to the moisture of 12.5%. AD was measured from planting to the day when 50% of the plants in a plot shed pollen. PH was measured from the soil surface to the flag leaf collar on five representative plants in each plot, and the average was used for the analysis.

### Genotypic Data

The 3,068 DH lines were panted in the greenhouse, and leaf samples were taken 2–3 weeks after emergency and sent to Intertek, Sweden, for DNA extraction. The DNA sample plates were forwarded to Cornell Life Science Core Laboratory Center, Ithaca, NY, the USA for genotyping with rAmpSeq markers described by Buckler et al. (2016). The rAmpSeq genotyping platform is based on the whole-genome sequences of repetitive sequences to identify polymorphisms using bioinformatics tools (Buckler et al., 2016, http://www.biorxiv.org/content/early/2016/12/24/096628). The rAmpSeq platform provides dominant markers, and a total of 9,155 markers coded as 0 (absence) and 1 (presence) were filtered by minor allele frequency (MAF<0.05), from which 5,173 were used for GS.

### Phenotypic Data Analysis

All the phenotypic analyses were done for each location and across locations within and across years to obtain the variance components and best linear unbiased estimates (BLUEs) described by Rezende et al. (2020). The BLUEs across locations for each trait were generated using the following linear mixed model using META-R software (Alvarado et al., 2020):

(1) $$\begin{array}{r} Y_{ijrk}=\mu +L_{j}+R_{r}\left(L_{j}\right)+B_{k}\left[R_{r}\left(L_{j}\right)\right]+G_{i}+GL_{ij}+\varepsilon_{ijrk} \end{array}$$

where *Y*_*ijrk*_ is the mean value of genotype *i* at location *j* in replicate *r* within the block *k*; *μ* is the general mean; *L*_*j*_ is the fixed effect of the location *j*; *R*_*r*_(*L*_*j*_) is the fixed effect of the replicate *r* within location *j*; *B*_*k*_[*R*_*r*_(*L*_*j*_)] is the random effect of the incomplete block *k* within replicate *r* and location *j* assumed to be independent and identically normally distributed with mean zero and variance $\sigma_{\;B\left(RL\right)}^{2}$; *G*_*i*_ is the fixed effect of genotype *i*; *GL*_*ij*_ is the fixed effect of the genotype × location interaction; and ε_*ijrk*_ is the random residual error assumed independent and identically normally distributed with mean zero and variance $\sigma_{\varepsilon }^{2}$.

Broad-sense heritability (H^2^) was estimated based on the entry mean according to:

(2) $$\begin{array}{r} H^{2}=\frac{\sigma_{g}^{2}}{\sigma_{g}^{2}+\;\frac{\sigma_{gl}^{2}}{l}+\;\frac{\sigma_{\varepsilon }^{2}}{lr}}\; \end{array}$$

where $\sigma_{g}^{2}$ is the genotype variance; $\sigma_{gl}^{2}$ is the genotype × location interaction variance; and $\sigma_{\varepsilon }^{2}$ is the error variance for *l* locations and *r* replicates of the genotypes at each site. The analysis across trials was performed using a similar model as those shown above and including the trial as a fixed effect.

### Genomic Prediction Models and Methods

The 3,086 DH lines (training set, TRN) were evaluated across locations under WW and WS. GEBVs were calculated for GY, AD, and PH using the BGLR statistical R-package (Pérez and de los Campos, 2014) within and across years for WW and WS sites using the BLUEs of entries within and across years. For genome-enabled prediction, a total of 5,173 markers that passed quality control were selected. For GS, the G-BLUP model was employed as follows:

(3) $$\begin{array}{r} y_{ij}=\mu +E_{i}+g_{j}+gE_{ij}+\;\varepsilon_{ij}, \end{array}$$

where *y*_*ij*_ is the response trait for the *j*th hybrid in the *i*th environment, *μ* is an intercept, and *E*_*i*_ is the random effect of the Environment (year-location-management combination, with management, is WW or WS), *E*_*i*_ are assumed to be normally and independently distributed with zero mean and variance parameter $\sigma_{E}^{2}$. Here, *g*_*j*_ represents a random additive effect of the *j*th line, we assume $\text{g}={\left(g_{1},\ldots ,g_{l}\right)}^{{\;}^{\prime }}$ follows a multivariate normal distribution with zero mean and variance–covariance matrix $\sigma_{g}^{2}\text{G}$, where *l* is the number of lines, **G** is a genomic relationship matrix computed using the matrix of marker genotypes centered and standardized by columns (Lopez-Cruz et al., 2015) and $\sigma_{g}^{2}$ is a variance parameter associated to lines. *gE*_*ij*_ represents the interaction between location *i* and hybrid *j*, we assume that **gE** = {*gE*_*ij*_} follows a multivariate normal distribution with zero mean and variance covariance matrix $\sigma_{gE}^{2}\left({\text{Z}}_{g}{\text{G}\text{Z}}_{g}\right)\#\left({\text{Z}}_{E}{\text{Z}}_{E}\right)$, where **Z**_*g*_ is a matrix that connects response variable with hybrids, **Z**_*E*_ is a matrix that connects the response variable with environments, $\sigma_{gE}^{2}$ is the variance parameter associated to the interaction between genotypes and environment and # represents the Hadamard product (cell by cell) between two matrixes. Finally, we assume that ε_*ij*_ are normal, independent, and identically distributed random variables with zero mean and variance $\sigma_{\varepsilon }^{2}$. The model described above corresponds to a reaction norm model which has been used in context of genomic prediction before (Jarquín et al., 2014).

### Cross-Validation and Prediction Accuracy Estimation

We used four scenarios for predictions. Scenario (i) data from 2017 is used as TRN set, and 2018 as a TST set, (ii) data from 2017 is used as a TRN set and predict 2019 data, (iii) data from 2018 was used as a TRN set and predict 2019 data and (iv) both 2017 and 2018 trait data were used as TRN set and 2019 as TST set. A cross-validation scheme with 20 replications was used to generate the TRN and TST sets and assess the prediction accuracy. In each of the 20 replications, the observations were randomly selected and assigned to the training (TRN) and testing (TST) sets. Furthermore, the TST population was randomly partitioned into 10, 30, 50, 70, and 90%, and the remaining 90, 70, 50, 30, and 10% of the TST population was combined with the corresponding TRN set and predicted the remaining TST populations (Supplementary Table 1).

## Results

### Variance Components and Broad-Sense Heritability

**Figure 1:**
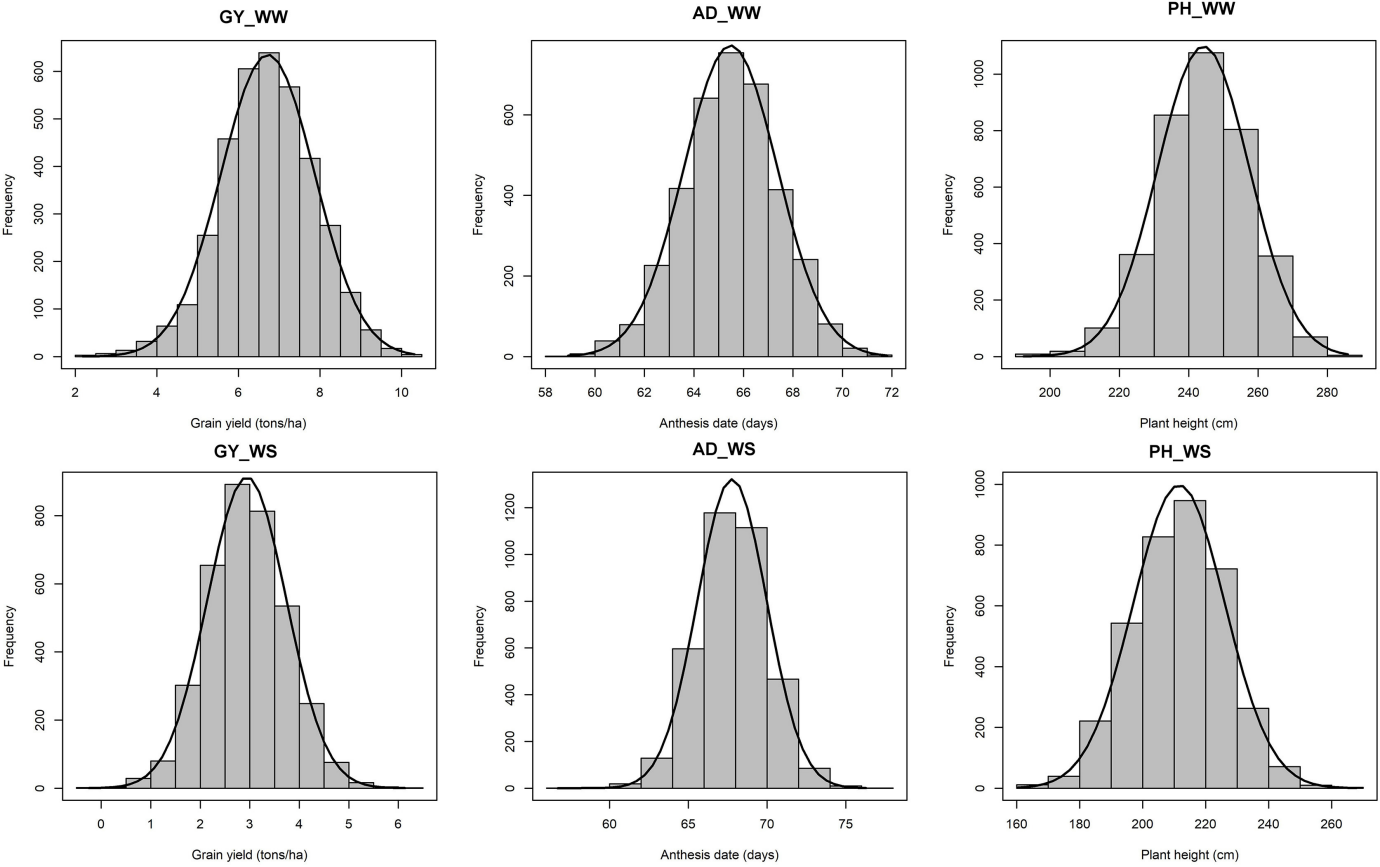
Phenotypic distribution of GY, AD, and PH under optimum (top) and managed drought (bottom) conditions. GY, grain yield; AD, anthesis date; PH, plant height.

All traits under WW and WS followed a normal distribution (Figure 1). Heritability of GY for each year ranged from 0.64 to 0.91 under WW and 0.17 to 0.50 under WS conditions (Table 1). Combined across the 3 years, heritability for GY was 0.91 under WW and 0.96 under WS. Under WW, the heritability of PH and AD for each year was high and ranged from 0.67 to 0.95 and 0.82 to 0.96, respectively, while under WS, it ranged from 0.23 to 0.57 and 0.57 to 0.67, respectively. Under WW, combined across 3 years, heritability was high for all traits and ranged from 0.91 to 0.96, while under WS, it ranged from 0.96 to 0.97 (Table 1). Under WW conditions, mean GY ranged from 6.05 t/ha in 2017 to 8.02 t/ha in 2018, while under WS, it ranged from 2.29 t/ha in 2019 to 3.28 in 2017. Drought stress reduced GY by 45.8, 61.2, and 63.9% in 2017, 2018, and 2019 seasons, respectively (Table 1), while PH was reduced by 11.4, 15.9, and 13.5% in 2017, 2018, and 2019, respectively.

**Table 1:** Estimation of variance components for grain yield and agronomic traits assessed in 3 years and across trials under optimum and drought conditions.

	**Optimum**			**Drought**			**Optimum**			**Drought**		
	**GY**	**AD**	**PH**	**GY**	**AD**	**PH**	**GY**	**AD**	**PH**	**GY**	**AD**	**PH**
**Year-2017**							**Year-2019**					
$\sigma_{\text{G}}^{2}$	0.17^**^	1.07^**^	34.30^**^	0.08^*^	1.94^**^	34.56^**^	0.12^**^	0.57^**^	17.10^**^	0.07^*^	1.39^**^	27.82^**^
$\sigma_{\text{T}}^{2}$	0.48	143.25	1072.14	-	-	-	0.24	115.35	538.31	-	-	-
$\sigma_{\text{GT}}^{2}$	0.00	0.00	1.08^**^	-	-	-	0.05^**^	0.05^**^	17.46^**^	-	-	-
$\sigma_{\text{e}}^{2}$	2.23	3.78	227.62	0.76	1.88	190.87	2.19	4.12	251.28	0.61	2.12	187.59
*H^2^*	0.68	0.89	0.81	0.17	0.67	0.27	0.64	0.82	0.67	0.18	0.57	0.23
Mean	6.05	64.24	236.04	3.28	63.41	209.03	6.34	65.75	247.28	2.29	67.48	213.87
CV (%)	7.98	1.57	2.57	10.95	1.79	3.42	7.11	1.32	2.15	14.60	1.64	3.07
LSD_0.05_	0.95	1.97	11.90	0.70	2.23	14.01	0.88	1.70	10.42	0.66	2.17	12.87
**Year-2018**							**Across 3 years**					
$\sigma_{\text{G}}^{2}$	0.36^**^	1.33^**^	63.68^**^	0.25	2.08	79.27	0.19^**^	0.80^**^	35.26^**^	0.12^**^	0.94^**^	40.82^**^
$\sigma_{\text{T}}^{2}$	1.11	149.85	39.00	-	-	-	0.00	83.11	509.71	0.25	21.97	0.00
$\sigma_{\text{GT}}^{2}$	0.21^**^	0.63^**^	30.58^**^	-	-	-	0.14^**^	0.59^**^	25.12^**^	0.04^*^	1.08^**^	12.46^**^
$\sigma_{\text{e}}^{2}$	1.91	2.68	172.82	0.50	2.40	117.84	2.10	3.33	210.71	0.61	2.05	159.48
*H^2^*	0.91	0.96	0.95	0.50	0.63	0.57	0.91	0.96	0.95	0.96	0.97	0.97
Mean	8.02	66.68	252.11	3.11	72.79	212.09	6.69	65.55	243.96	2.89	67.90	211.91
CV (%)	8.58	1.75	3.29	16.24	1.76	3.91	8.07	1.55	2.83	14.41	1.68	3.56
LSD_0.05_	1.35	2.29	16.28	0.99	2.51	16.26	1.06	1.99	13.53	0.82	2.24	14.77

*GY, grain yield; AD, anthesis date; PH, plant height*.

* *and*

** *indicate significance at P < 0.05 and P < 0.01, respectively*.

### Prediction Accuracy of 1-Year Breeding Data From the Independent Validation Schemes of Another Year of Breeding Data

**Figure 2:**
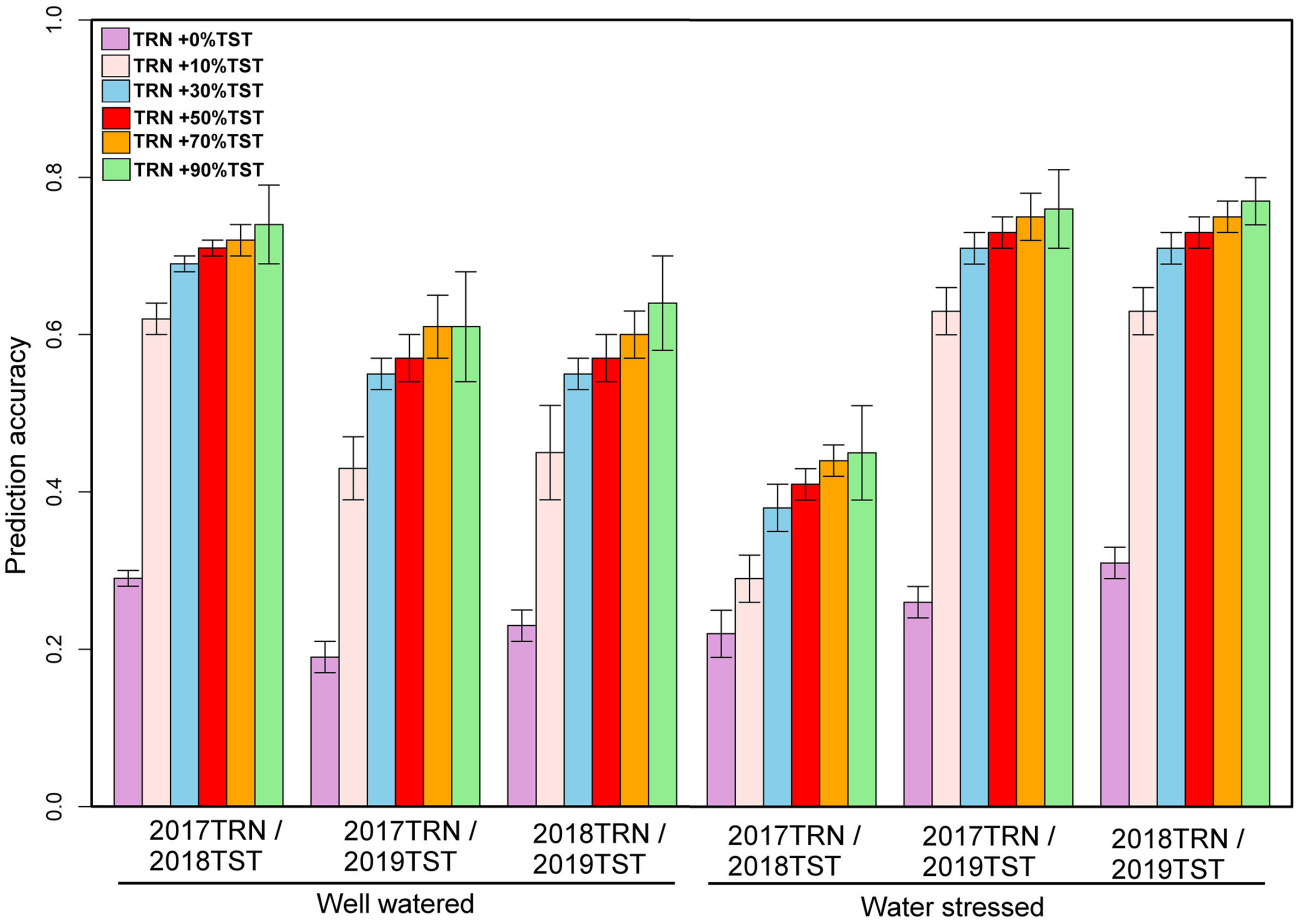
Prediction accuracies for GY estimated using 1-year data to predict another year's data and converting 10, 30, 50, 70, and 90% of the data from the testing population to the training population under WW and WS conditions. GY, grain yield; WW, well-watered; WS, water-stressed.

**Figure 3:**
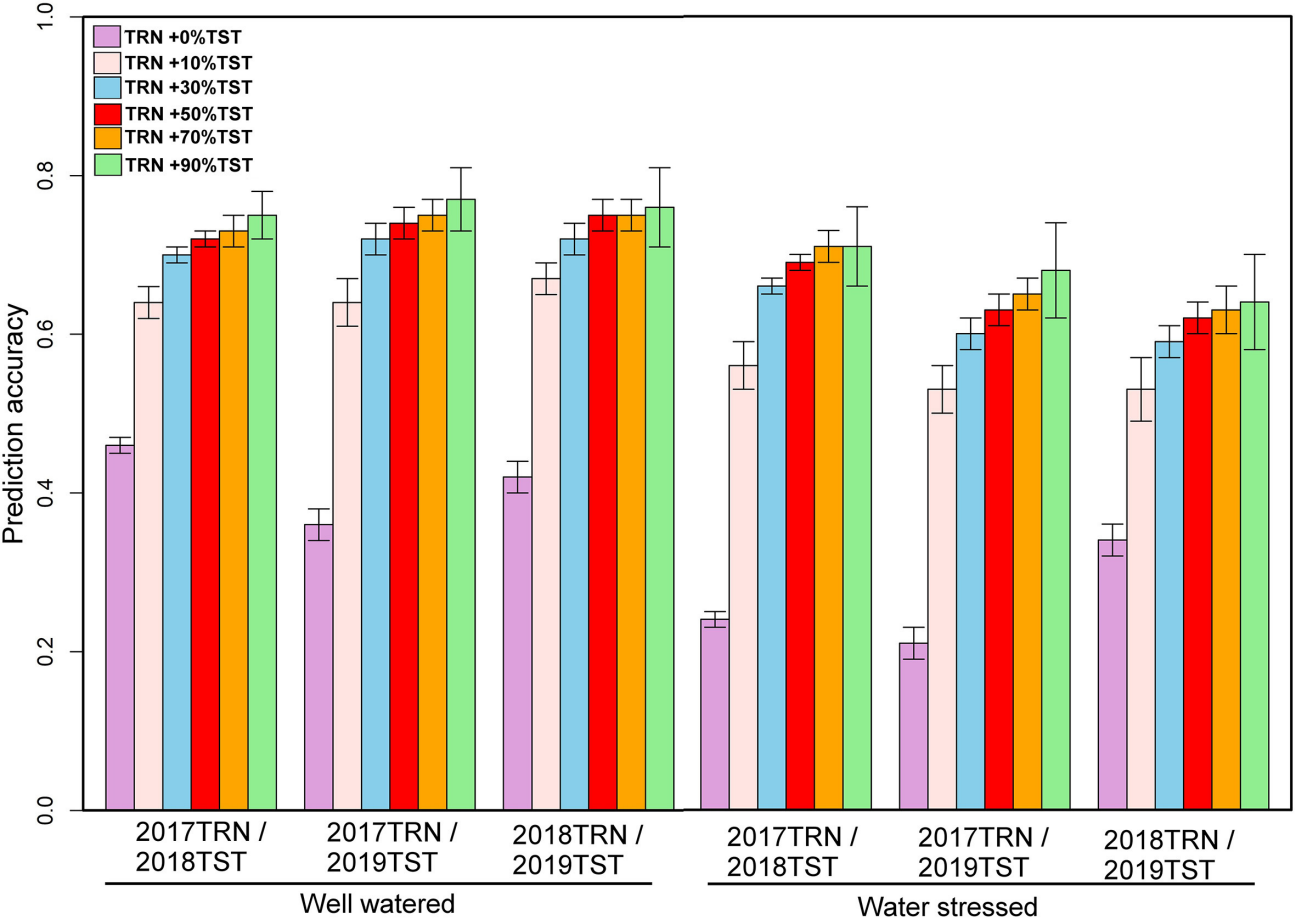
Prediction accuracies for anthesis date estimated from independent validation schemes using 1-year data to predict another year's data and converting 10, 30, 50, 70, and 90% of the data from the testing population to the training population under optimum and managed drought conditions.

**Figure 4:**
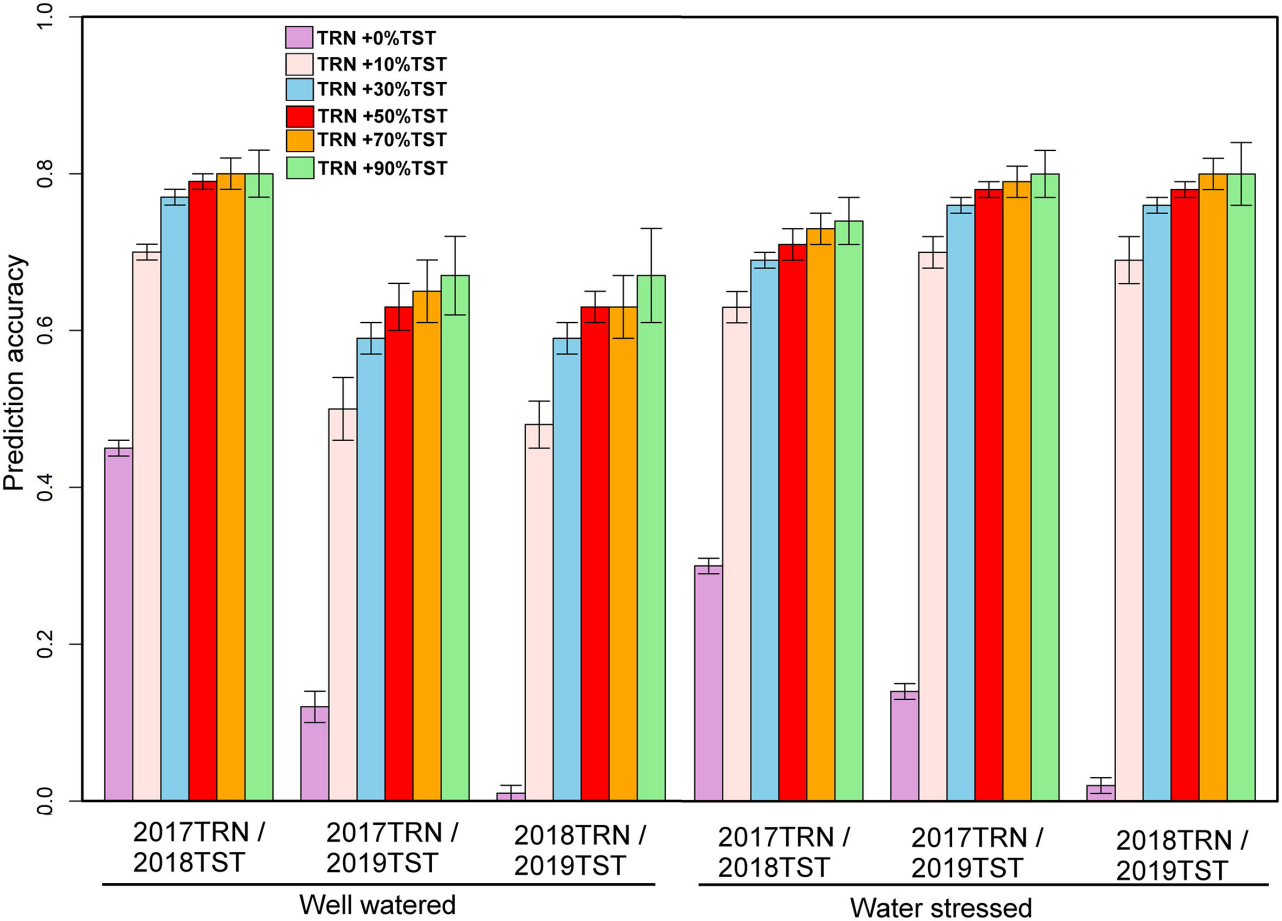
Prediction accuracies for plant height estimated from independent validation schemes using 1-year data to predict another year's data and converting 10, 30, 50, 70, and 90% of the data from the testing population to the training population under optimum and managed drought conditions.

The genotypic matrix of 3068 DH lines is depicted in Supplementary Figure 1. As shown in Supplementary Figure 1, there was some overlap between the three datasets, which suggests that individuals from a 1-year dataset can be predicted using the other year data set. The prediction accuracy of 1-year performance data using data from another year varied across traits and management conditions, as summarized in Figures 2–4. The predictive ability of GY using 1-year data to predict performance in a separate year ranged from 0.19 to 0.29 under WW and 0.22 to 0.31 under WS (Figure 2). Under WW, the predictive ability for AD ranged from 0.36 to 0.46, while for PH, it ranged from 0.01 to 0.45. Under WS, the predictive ability for AD ranged from 0.21 to 0.34, and for PH, it ranged from 0.01 to 0.30 (Figures 3, 4).

### The Predictive Ability of 1-Year Data by Using a Certain Proportion of Individuals From the Prediction Set as Part of the Training Set

In addition to using 1-year data to predict the other year data, we also converted 10, 30, 50, 70, and 90% of the data from the prediction set to the training set to understand the impact on prediction accuracy. With 2017 data combined with 10% of the data from 2018 and predicting the remaining 90% of 2018 data revealed an increase in prediction accuracy to 0.62 and 0.29 for GY; 0.64 and 0.56 for AD; and 0.70 and 0.63 for PH under WW and WS conditions, respectively (Figures 2–4). Similarly, while predicting 90% of 2019 data by using 2017 data and 10% of 2019, data revealed an accuracy of 0.43 and 0.63 for GY; 0.64 and 0.53 for AD; and 0.50 and 0.70 for PH under WW and WS, conditions, respectively (Figures 2–4). Prediction of 90% of 2019 data by using 2018 data combined with 10% of 2019 data showed an accuracy of 0.45 and 0.63 for GY; 0.67 and 0.53 for AD; and 0.48 and 0.69 for PH under WW and WS conditions, respectively (Figures 2–4). Incorporating the proportion of 30, 50, 70, and 90% of lines from the year of testing to TRN set showed a gradual increase in accuracy for GY from 0.69 to 0.74 under WW and from 0.38 to 0.45 under WS conditions. Similarly, accuracy was increased from 0.64 to 0.75 for AD and 0.70 to 0.80 for PH under WW conditions, while under WS conditions, accuracy was increased from 0.56 to 0.71 for AD and 0.63 to 0.74 for PH (Figures 3, 4). Prediction accuracy for 2019 data by using 2018 data and converting a certain proportion of 2019 data also revealed an increase in accuracy for GY from 0.55 to 0.64 under WW and from 0.71 to 0.77 under WS conditions.

High prediction accuracy for GY across WW and WS environments was obtained when using 1-year data in combination with 30% data from the year of testing, whose predictive ability ranged from 0.55 to 0.69 and 0.38 to 0.71, respectively—much higher than those trained in individual years. Integrating 50, 70, and 90% of the data from the TST set to the TRN set did not significantly increase the prediction accuracy for GY compared to integrating 30% of the data from TST to TRN set in all years (Figure 2). The prediction accuracy for agronomic traits (AD and PH) across WW and WS environments obtained using 1-year data and integrating 10, 30, 50, 70, and 90% of the data from TST set to TRN set under both WW and WS conditions was much higher than those trained in individual years.

### Predictive Ability of Pooled Data and Integrating a Certain Proportion of Individuals From the Prediction Set as Part of the Training Set

**Figure 5:**
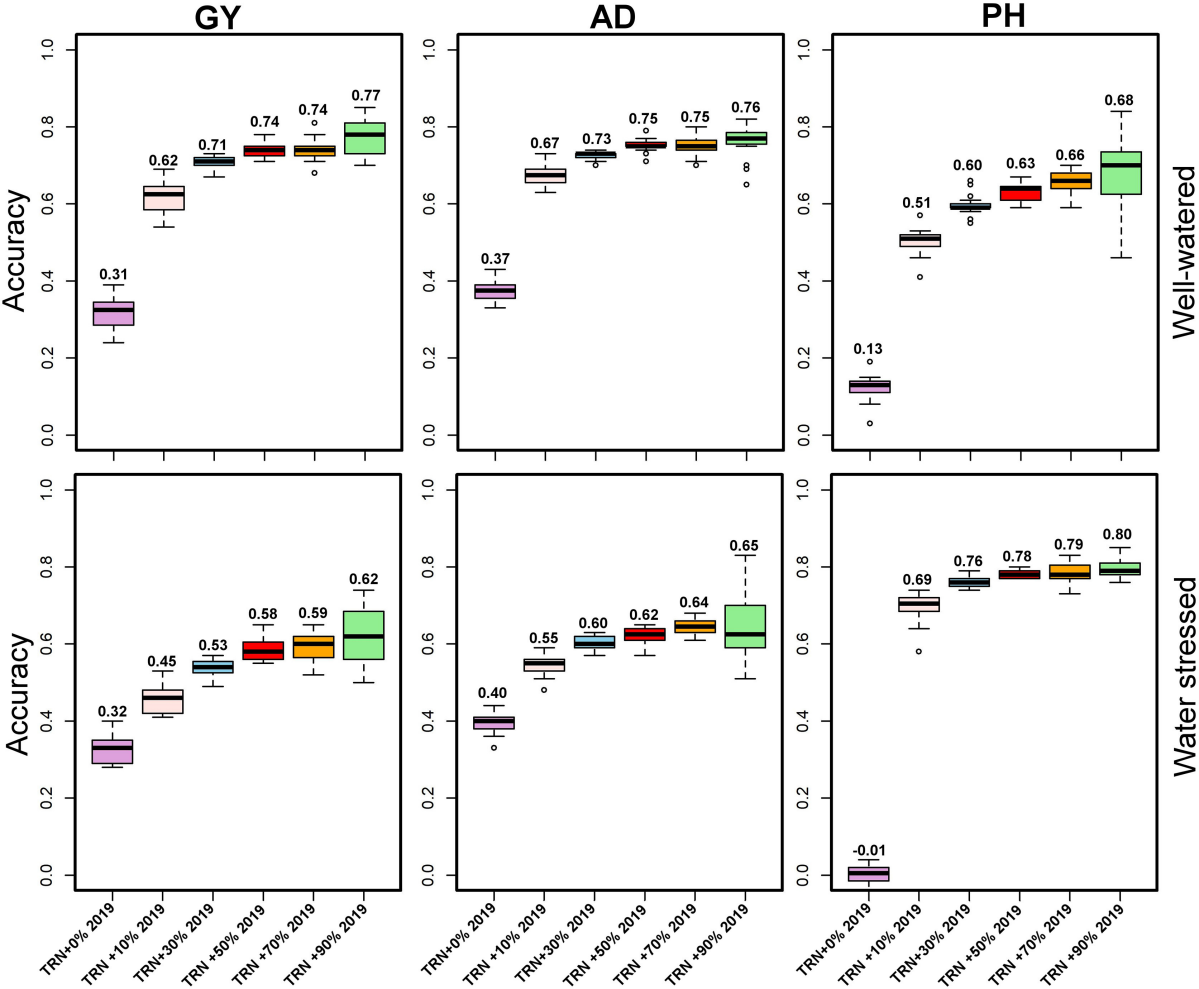
Prediction accuracies for grain yield and agronomic traits assessed estimated from independent validation schemes using a training population (TRN) consisting of 2017- and 2018-years breeding data and 10, 30, 50, 70, and 90% of 2019 data converted from the testing population (TST) to the training population under optimum and managed drought conditions. GY, grain yield; AD, anthesis date; PH, plant height.

Across-year predictive ability was investigated using pooled data (2017+2018), and the predicting 2019 data performance showed a slight improvement (0.32) for GY under WW and 0.31 under WS (Figure 5). While prediction accuracy for AD was 0.37 (WW) to 0.40 (WS), for PH, it was 0.01 under WS to 0.13 under WW (Figure 5). The prediction accuracies were further improved by integrating 10, 30, 50, 70, and 90% of the TST set to the TRN set (Figure 5). Using 2 years' data (2017 and 2018) and integrating 10% of 2019 data to the TRN set, the prediction accuracy for GY was increased to 0.45 under WW and 0.62 under WS conditions. Using pooled data and incorporating 30, 50, 70, and 90% of 2019 data to TRN set, the mean prediction accuracy for GY was 0.74 and ranged from 0.71 to 0.77 under WS, while the prediction accuracy was 0.58 and ranged from 0.53 to 0.62 under WW conditions. The mean prediction accuracy for AD was 0.61 under WS and 0.73 under WW by using pooled data and integrating 10, 30, 50, 70, and 90% of the data from TST to TRN set.

## Discussion

Maize improvement in the tropics has been successful in improving grain yield under stress and non-stress conditions and has contributed to the food security and livelihoods of smallholder farmers (Renkow and Byerlee, 2010; Krishna et al., 2021; Prasanna et al., 2021). To keep up with the growing food demand, new tools and technologies must be used to increase genetic gains, especially in stress-prone tropical environments (Prasanna et al., 2021). Reducing cycle time is one of the key factors responsible for increasing genetic gains in crop breeding programs without greatly increasing the program size (Atlin et al., 2017; Cobb et al., 2019). Reduction of breeding cycle time can be achieved by recycling the lines at an earlier stage as breeding parents, coupled with the implementation of GS to improve selection accuracy when selecting breeding parents with fewer years of phenotypic data available.

CIMMYT Global Maize Program has evaluated various strategies to implement GS in maize breeding pipelines with promising results (Beyene et al., 2015, 2019; Ceron-Rojas et al., 2015; Vivek et al., 2017; Wang et al., 2020). Crossa et al. (2017) reported that GS is better than phenotypic selection to reduce breeding cycles and operational costs. Beyene et al. (2015) reported that the average gain from the rapid cycle of GS across eight populations was 0.086 Mg ha^−1^.

In this study, we used large data sets of 3,068 DH lines genotyped and phenotyped as test crosses across 3 years in WW and WS conditions in Kenya to evaluate the potential of genomic prediction using existing breeding data from previous years to predict untested lines at an early testing stage to bypass the first-year phenotyping stage, saving a year in the process. This study showed that the average prediction accuracies were 0.32 under WW and 0.31 under WS conditions when the pooled 2-year datasets were used as TRN to predict the third-year data set. Similar results were also reported by Wang et al. (2020) using data from the CIMMYT Latin America breeding program, where the average prediction accuracies ranged from 0.31 to 0.42 when 2-year datasets were used as TRN to predict the third year TST set data. Beyene et al. (2019) reported higher prediction accuracy for GY (0.67 under well-watered and 0.65 under managed drought conditions) using the training and prediction set within a single year (phenotyping half and predicting half). The same study also showed that the performance of lines advanced using GEBVs or phenotypic values showed similar performance in the second stage of field testing under optimum and managed drought conditions. Inclusion of full-sib families in the TRN set has previously been shown to increase prediction accuracy when compared with prediction where no full-sib families are included (Brauner et al., 2020), consistent with the finding in this study that prediction accuracy improves when including a sample of selected candidates from within the group of candidates being predicted. Additionally, the testing year environments for the materials being predicted in the testing set are represented within the TRN phenotypic data set, contributing to an increase in the prediction accuracy when using 5-fold validation since the independent year and pooled year TRN sets alone by nature do not sample environments in the TST set.

One problem when using historical data is the limited connectivity of selection candidates evaluated across multiple years. To improve prediction accuracy, we integrated a certain proportion of the lines from the prediction set to the training set. Results showed that by using 1-year data and merging 10% of data from the year of testing to TRN, a mean prediction accuracy for GY was increased to 0.52 under WS and 0.50 under WW conditions. Using 1-year data combining with 30% of data from the year of testing, the predictive ability for GY increased further (0.60 under WS and 0.59 under WW) more than model training in the individual year. Integrating 50, 70, and 90% of the data from the year of testing to TRN demonstrated diminishing returns in terms of increased prediction accuracy for GY (mean 0.64 under WS and WW) compared to integrating 30% of the data from TST to TRN in all years (Figures 3–5). These results conformed with those from the previous studies, which indicated that prediction accuracy could be improved by strengthening the relationship between the training and prediction sets (Daetwyler et al., 2013; Crossa et al., 2017; Zhang et al., 2017; Brandariz and Bernardo, 2019; Atanda et al., 2021). Our results demonstrated that using a training set from existing variety development pipelines that had genotypic and phenotypic data is useful in the routine implementation of GS. This approach will significantly reduce the cost of performing test crosses and field evaluation of large numbers of stage 1 data in the breeding pipelines. As shown in Beyene et al. (2019), when the objective is to discard lines with poor breeding values from advancing to resource-demanding multi-location yield trials, moderate genomic prediction accuracy should suffice without losing selection accuracy.

Benefits of genomic prediction using historical datasets from the ongoing variety development pipelines as training sets have been reported (Wang et al., 2020; Atanda et al., 2021). Gaynor et al. (2017) reported that GS reduced cycle time from 4 to 3 years per cycle of genetic improvement compared to a conventional breeding scheme without GS, even though it may be less accurate than phenotypic selection. In a forward prediction scenario, good predictive abilities (0.53 to 0.71) were observed in our study when the TRN consists of breeding data from two previous years and 30% of data from a third year's data than using individual year-based predictions. The observed predictive ability is promising, especially under drought, considering the low heritability and that the training set differed from those sets used to evaluate the prediction set. Beyene et al. (2019) compared GS to phenotypic selection in CIMMYT's eastern Africa maize breeding program and reported that there was no significant difference between the mean of hybrids advanced through phenotypic and GS both under optimum and managed drought stress conditions, but that GS reduced the cost by 32% over PS. This strategy consisted of testing half and predicting the remaining half based on marker data currently being implemented in CIMMYT's eastern Africa and Latin America breeding hubs (Beyene et al., 2019; Wang et al., 2020). However, the use of historical data in this study suggests that this strategy can be further refined to test 10–30% and predict the remaining 70–90% of the lines, using the historical data also as a part of the TRN in the predictions. This will save resources without affecting the selection accuracy. Further integration of different designs like sparse testing and/or partially replicated trials can further help evaluate the selected 10–30% of lines tested in more environments to get the high-quality data with the same amount of resources. Using strong historical data, the evaluation step of eliminating early-stage breeding lines is an objective of a long-term breeding goal. Nevertheless, with 3 years of historical data, based on the results from this study, we propose to reduce the TRN from the current 50 to 10–30% in the future, especially as the historical data increases; more related lines joining this can help to fulfill this long-term goal of eliminating the phenotyping of whole early-stage testing and extending it to other breeding pipelines.

If the primary goal is to reduce the breeding cycle time and accelerate the identification of elite new hybrids, a practical strategy change could be made to the breeding program design to take advantage of the lower prediction accuracies using pooled historical data simply by reallocating resources from the large and expensive first-year testing scheme into an expanded second-year test equivalent trialing system. In the traditional breeding scheme used before 2018, ~65–70% of the phenotyping costs of the Stages 1 and 2 trial system were spent on Stage 1 evaluation, while in the test-half-predict-all scheme, this was reduced to around 50%. Although further reduction of the Stage 1 trials component to 35% of the total Stages 1 and 2 trial system can deliver cost savings while maintaining relatively similar prediction accuracy. If the primary goal is to reduce cycle time rather than reduce overall cost, then the lower prediction accuracy using historic data could be offset by eliminating the Stage 1 phenotyping costs and testing more candidates selected on GEBVs in Stage 2 equivalent trials. It would be possible to double the number of selection candidates advanced based on GEBV using historical data within our current budgets instead if no selection candidates were being evaluated in Stage 1 equivalent trials. Although we would expect the cohort of materials advancing into Stage 2 equivalent trials each year to have a lower mean performance value because of the reduction in prediction accuracy, the likelihood of identifying the best new lines from each population could be improved simply by testing more of them. This simple strategy shift at cost equivalency would shorten both the breeding cycle time and market with elite new varieties. Furthermore, continued expansion of the multi-year TRN data will enable further refinement and improvement of prediction accuracy as this will enable more effective predictions within heterotic groups or sub-groups within a heterotic group and across product-profile-based breeding pipelines.

## Conclusions

Genomic selection could help predict the breeding value of newly developed DH lines for the next stage of testing. Prediction by using historical data alone yielded relatively low accuracy for GY and other agronomic traits. However, by combining only 10% to 30% of the lines from the year of testing, we could achieve a significant increase in prediction accuracy. CIMMYT's Eastern Africa maize breeding program is currently implementing GS at an early stage of testing using a test-half-predict-all strategy, as Beyene et al. (2019) described. With the historical data, we can reduce the current training population size from 50 to 10–30% to achieve the same or even higher level of accuracy. This could save costs associated with the testcross formation and multi-location evaluation. Nevertheless, the time required for completion of the breeding cycle remains the same, as the limited historical data available from one breeding program with one or two seasons of data provides prediction accuracy of approximately half of what can be achieved with the inclusion of some full sibs in the training set. Nevertheless, the time required to complete the breeding cycle remains the same, as historical data alone does not yield promising accuracy both under optimum and drought stress conditions. However, with careful planning, it is possible to skip the whole stage I testing with only historical data in predictions. There is a need to have some proportion of parental lines shared between the historical data set and the current prediction set. Further research is warranted to know what proportion of lines should be common between historical data and prediction. However, we theorize that even with the modest prediction accuracies found when using one or 2 years of historical data within a single breeding program, the use of GS using only historical data to advance a larger number of selected candidates directly to a Stage 2 equivalent trial should yield similar overall performance gains from the breeding program at the same cost with 1 year lesser time. Further, it seems plausible that prediction accuracies when using historic data could improve by the inclusion of additional years of data assuming reasonable genetic relationships across cohorts of selection candidates over time.

## Data Availability Statement

The original contributions presented in the study are included in the article/Supplementary Material, further inquiries can be directed to the corresponding authors.

## Author Contributions

YB, MG, JC, PP, MO, BP, KR, and JB were responsible for planning the experiment. YB was responsible for developing the populations and conducting field trials. MG was responsible for genotyping the lines used in the study. PP, JC, MG, and YB carried out all the phenotyping and genotypic analysis. YB, JC, MO, and MG wrote the manuscript. All authors have contributed to the final version.

## Conflict of Interest

The authors declare that the research was conducted in the absence of any commercial or financial relationships that could be construed as a potential conflict of interest.

## Supplementary Material

Supplementary Figure 1 Three-dimensional plot based on three eigenvectors of genotypic data to show the genetic relationship among the 3 years data set used in the study.

Supplementary Table 1 Training (TRN) and testing (TST) sets the composition used for each prediction scenario.
