# Supplementary figures and images for: Application of Genomic Selection at the Early Stage of Breeding Pipeline in Tropical Maize
Source: Front Plant Sci. 2021 Jun 28;12:685488. doi: 10.3389/fpls.2021.685488 (PMC8274566; doi:10.3389/fpls.2021.685488)

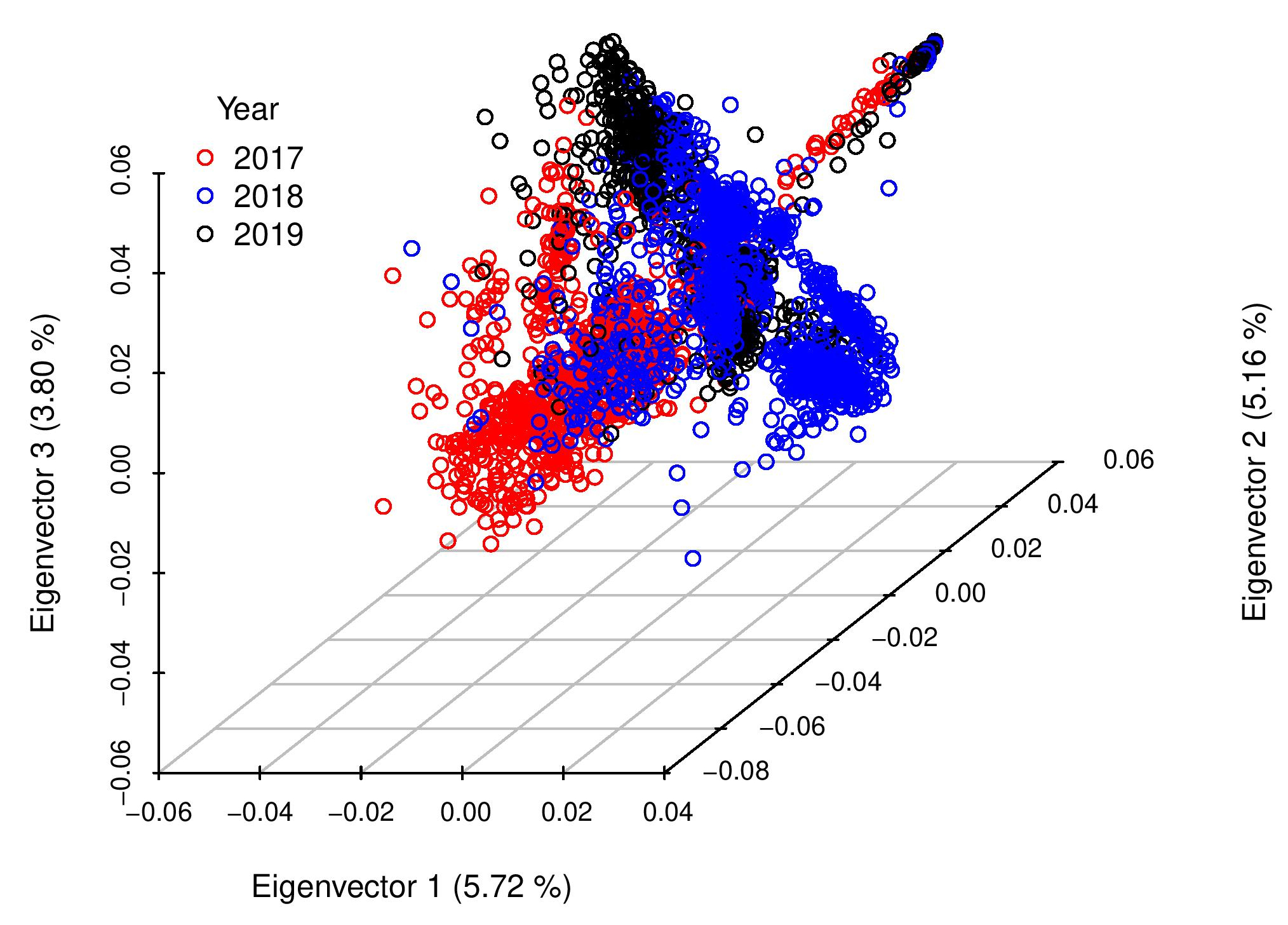

Supplement: Supplementary Figure 1 — Three-dimensional plot based on three eigenvectors of genotypic data to show the genetic relationship among the 3 years data set used in the study. [file Image_1.JPEG]
