# Supplementary material for: Application of Genomic Selection at the Early Stage of Breeding Pipeline in Tropical Maize
Source: Front Plant Sci. 2021 Jun 28;12:685488. doi: 10.3389/fpls.2021.685488 (PMC8274566; doi:10.3389/fpls.2021.685488)
Supplement: Supplementary Table 1 — Training (TRN) and testing (TST) sets the composition used for each prediction scenario. [file Table_1.DOCX]

**Supplemental Table 1**. Training (TRN) and testing (TST) sets composition used for each prediction scenario.

| Scenario 1 | 2017 data (N) | Training set (TRN): 2017 data + x % of 2018) | Testing set (TST): (100-x) % 2018 |
| --- | --- | --- | --- |
|  | 923 | 923 (0%) | 1423 |
|  |  | 1065 (10%) | 1281 |
|  |  | 1035 (30%) | 996 |
|  |  | 1635 (50%) | 712 |
|  |  | 1919 (70%) | 427 |
|  |  | 2204 (90%) | 142 |
| Scenario 2 | 2017 data (N) | Training set: 2017 data + x % of 2019) | Testing set: (100-x) % 2019 |
|  | 923 | 923 (0%) | 722 |
|  |  | 995 (10%) | 650 |
|  |  | 1140 (30%) | 505 |
|  |  | 1284 (50%) | 361 |
|  |  | 1428 (70%) | 217 |
|  |  | 1773 (90%) | 117 |
| Scenario 3 | 2018 data (N) | Training set: 2018 data + x % of 2019) | Testing set: (100-x) % 2019 |
|  | 1423 | 1423 (0%) | 722 |
|  |  | 1495 (10%)) | 650 |
|  |  | 1640 (30%) | 505 |
|  |  | 1780 (50%) | 361 |
|  |  | 1928 (70%) | 217 |
|  |  | 2073 (90%) | 117 |
| Scenario 4 | 2017+ 2018 pooled data(N) | Training set: 2017 + 2018 + x % of 2019 | Testing set: (100-x) % 2019 |
|  | 2346 | 2346 (0%) | 722 |
|  |  | 2418 (10%) | 650 |
|  |  | 2563 (30%) | 505 |
|  |  | 2707 (50%) | 361 |
|  |  | 2851 (70%) | 217 |
|  |  | 2996 (90%) | 117 |
